# KAT2A-IGF2BP1-CXCL2 axis in the high lactate tumor microenvironment facilitates resistance to anti-PD-1 therapy in lung adenocarcinoma by recruiting myeloid-derived suppressor cells

**DOI:** 10.1038/s41419-026-09018-z

**Published:** 2026-06-25

**Authors:** Gen Li, Chunping Wang, Ao Wang, Haiyang Wang, Chuanliang Peng

**Affiliations:** 1 Department of Wound Reconstructive Surgery, Tongji Hospital, School of Medicine, Tongji University, Shanghai, China; 2 Department of Thoracic and Cardiovascular Surgery, Tongji Hospital of Tongji University, School of Medicine, Tongji University, Shanghai, China; 3 Department of Pathology, Shanghai Public Health Clinical Center, Fudan University, Shanghai, China; 4 Department of Laboratory Medicine, Tongji Hospital of Tongji University, School of Medicine, Tongji University, Shanghai, China; 5 Department of Thoracic Surgery, Shandong Cancer Hospital and Institute, Shandong First Medical University and Shandong Academy of Medical Sciences, Jinan, China; 6 Tongji Hospital, School of Medicine, Tongji University, Shanghai, China

**Keywords:** Non-small-cell lung cancer, Immunoediting

## Abstract

Anti-PD-1 therapy has significantly improved the clinical outcomes of patients with advanced lung adenocarcinoma (LUAD). However, primary and acquired drug resistance remain common and are largely driven by an immunosuppressive tumor microenvironment (TME). Lactate accumulation has emerged as a key metabolic determinant that shapes immunosuppressive niches. However, how lactate-derived epigenetic modifications regulate tumor immune escape and resistance to anti-PD-1 therapy in LUAD remains unclear. Here, we revealed that the KAT2A-IGF2BP1-CXCL2 axis in LUAD mediated recruiting myeloid-derived suppressor cells (MDSCs) and PD-1 blockade resistance. Clinically, pan-lactylation levels are significantly elevated in anti-PD-1-resistant LUAD samples and are correlated with high cell proliferation and elevated lactate levels in the tumor microenvironment. Single-cell transcriptomics revealed that KAT2A was enriched in epithelial cells and tumor-associated macrophages (TAMs) of therapy-resistant cases, where KAT2A was linked to immunosuppression. It has been further confirmed that KAT2A promoted lactylation of the N6-methyladenosine (m⁶A) reader IGF2BP1 at site K228 in vivo and in vitro experiments. This modification enhances IGF2BP1’s binding to the m⁶A site within CXCL2 mRNA, thereby increasing CXCL2 stability. KAT2A facilitated resistance to anti-PD-1 therapy in LUAD, inhibited the recruitment of CD8^+^ T cells, and significantly increased the infiltration of MDSCs. Furthermore, animal experiments demonstrated that targeted inhibition of KAT2A activity markedly enhanced the sensitivity of LUAD to anti-PD-1 therapy. This study provided a comprehensive exploration of KAT2A’s oncogenic functions. It suggests a novel strategy to enhance the efficacy of anti-PD-1 therapy by inhibiting the KAT2A-IGF2BP1-CXCL2 signaling axis in LUAD.

## Introduction

Lung cancer remains the leading cause of cancer-related morbidity and mortality worldwide, with lung adenocarcinoma (LUAD) representing the most prevalent histological subtype [1]. Immune checkpoint inhibitors (ICIs), particularly antibodies targeting programmed cell death protein 1 (PD-1), have significantly improved clinical outcomes for certain subsets of patients with non-small cell lung cancer (NSCLC), including those with advanced or metastatic disease receiving first-line therapy and those undergoing neoadjuvant treatment for resectable tumors. However, the resistance to anti-PD-1 therapy severely adversely affects patient outcomes, and is a significant challenge for researchers and clinicians [2, 3]. A growing body of evidence now underscores immunosuppressive reprogramming of the tumor immune microenvironment, induced by factors such as hypoxia, specific cytokines, metabolic remodeling, and microbial activity, collectively contributes to enhanced tumor immune evasion and thereby confers resistance to anti-PD-1 therapy [4]. Within this context, tumor cells recruit and reprogram tumor-associated macrophages (TAMs), which become the most abundant and plastic immune cells in the tumor microenvironment (TME) [5]. These TAMs then orchestrate immunosuppression by promoting immune evasion, suppressing antigen presentation, modulating inflammation, and driving T-cell exhaustion. Numerous studies have demonstrated that TAMs abundance, polarization state, and spatial distribution strongly correlate with disease progression and immunotherapeutic resistance [6]. Immunosuppressive TAMs subsets, such as M2-like macrophages, impair antitumor immunity by secreting chemokines, soluble mediators, and metabolic suppressors that inhibit the infiltration and activation of CD8^+^ T cells [7, 8]. Thus, elucidating the regulatory mechanisms governing TAMs behavior within the LUAD TME, and defining their contributions to anti-PD-1 resistance represent critical challenges in modern tumor immunology.

Lactate-derived lactylation, a recently identified post-translational modification (PTM), has emerged as a key driver of immunosuppressive TME formation. Due to enhanced glycolysis and rapid proliferation, cancer cells accumulate large quantities of lactate, creating a highly acidic and lactate-enriched metabolic niche [9]. Lactate, once regarded merely as a metabolic waste product, is now recognized as a key regulator in the TME. Lactate in this environment has been shown to possess various biological functions that actively shape immunosuppression, thereby mediating tumor immune escape. It facilitates the recruitment of immunosuppressive leukocytes and drives macrophages toward an inhibitory polarization state. Additionally, it impairs antigen presentation by dendritic cells and dampens CD8⁺ T cell cytotoxicity [10]. Importantly, elevated lactate levels correlate with anti-PD-1 resistance, while blocking lactate production or increasing lactate clearance can restore antitumor immunity. Lysine lactylation (Kla) has emerged as a novel post-translational modification, establishing a direct functional link between metabolic states and gene expression control [11]. Kla modulates chromatin accessibility, transcriptional activity, and immune cell function [12–14]. However, its specific role in driving immunotherapy resistance in LUAD remains elusive.

N6-methyladenosine (m⁶A), a predominant post-transcriptional modification of messenger RNAs, plays a pivotal role in modulating the immune microenvironment of lung cancer and therapeutic resistance [15]. m⁶A can regulate mRNA stability, splicing, translation, and degradation, thereby influencing tumor proliferation, stemness, immune evasion, and the recruitment of suppressive myeloid cells [16, 17]. Among m⁶A regulators, the IGF2BP family members (IGF2BP1, IGF2BP2, and IGF2BP3), functioning as m⁶A readers, play a critical role in stabilizing oncogenic transcripts [18], while the mechanisms underlying the lactylation of IGF2BPs remain unclear. A comprehensive study on the regulation and function of IGF2BPs may therefore provide better insights into improving tumor therapy and preventing metastasis.

In this context, we systematically investigated how lactylation and m⁶A modification synergistically mediate anti-PD-1 resistance in LUAD. We first observed markedly elevated pan-lysine lactylation levels in anti-PD-1-resistant groups. Subsequently, we analyzed the cellular landscape of samples that exhibited resistance to anti-PD-1 therapy and found a significant infiltration of KAT2A-positive TAMs (KAT2A^+^ TAMs) within these cases. Furthermore, analysis of single-cell datasets from LUAD revealed that KAT2A is specifically highly proportionally expressed in epithelial cells and macrophages of tumor samples, underscoring its cell-type-specific expression pattern in the TME. Clinical analysis, in conjunction with both in vitro and in vivo experiments, indicated that KAT2A in LUAD promotes resistance to anti-PD-1 therapy and enhances the recruitment of myeloid-derived suppressor cells (MDSCs) into the LUAD microenvironment by upregulating the secretion of CXCL2. We subsequently investigated the molecular mechanisms by which KAT2A regulates CXCL2 expression and found that this regulation depends on IGF2BP1. Furthermore, animal experiments demonstrated that targeted KAT2A inhibition markedly increased LUAD tumor sensitivity to anti-PD-1 therapy. In summary, our work defines a cooperative mechanism between lactylation and m⁶A modification that promotes anti-PD-1 resistance in LUAD. Specifically, we demonstrate that KAT2A-mediated lactylation of IGF2BP1-K228 enhances CXCL2-driven MDSCs recruitment to maintain the immunosuppressive TME. Our findings provide mechanistic insights into how metabolic reprogramming fuels immunotherapy resistance and pinpoint the KAT2A-IGF2BP1-CXCL2 axis as a promising therapeutic target for enhancing immunotherapy outcomes in LUAD.

## Results

### Pan-lysine lactylation and the cellular landscape correlate with anti-PD-1 response in non-small cell lung cancer

**Fig. 1:**
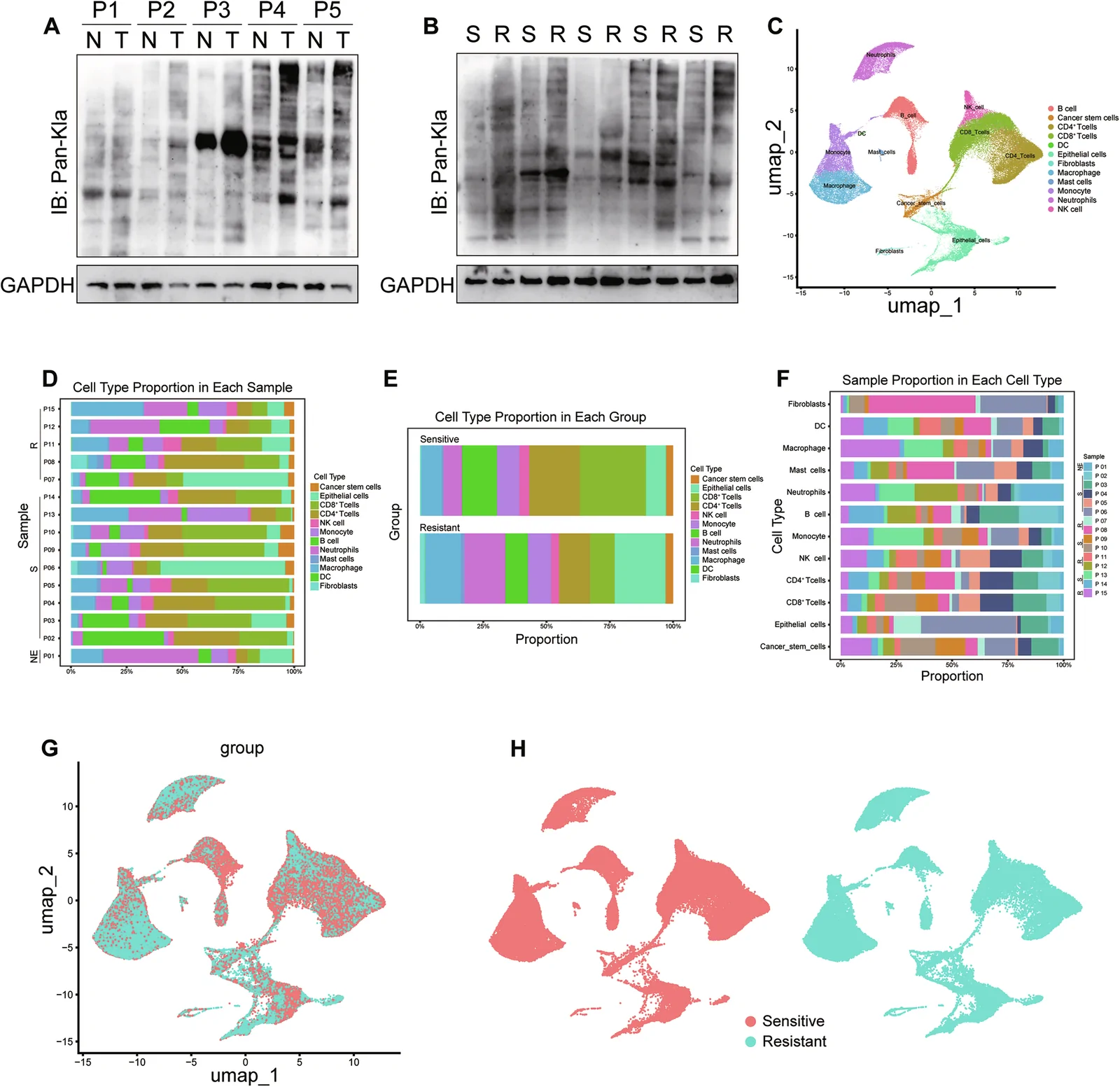
Pan-Kla expression levels and cellular landscape in the resistant and sensitive groups of anti-PD-1 therapy. **A** Western blot analysis of pan-Kla expression levels in tumor tissue (T) and adjacent non-cancerous tissue (N) from LUAD cancer patients. **B** Western blot analysis of pan-Kla expression levels in anti-PD-1-sensitive (S) and -resistant (R) tissue from LUAD patients. **C** Uniform manifold approximation and projection (UMAP) plot of all cells colored by major cell types according to canonical markers. **D** The proportions of cell clusters in each patient represented different cellular components in LUAD samples (sensitive group: S, resistant group: R, NE: Not Evaluated). **E** The proportion of macrophages in the anti-PD-1-resistant group was significantly higher than that in the sensitive group. **F** Distributions of different types of cells in the 15 LUAD patients. **G** UMAP visualization of cells in patients with different responses to anti-PD-1 therapy. **H** UMAP visualization of single-cell from anti-PD-1-sensitive (red) and resistant (blue) patients.

To investigate differences in pan-Lysine lactylation modifications between anti-PD-1-resistant and anti-PD-1-sensitive LUAD tissues, we collected tumor specimens from 10 patients who underwent surgical resection after receiving 2–4 cycles (3 weeks per cycle) of neoadjuvant therapy (PD-1 antibody + platinum-based chemotherapy). Western blot analysis revealed markedly elevated pan-Lysine lactylation levels in tumor tissue compared to adjacent non-cancerous tissue (Fig. 1A). Among the 10 patients, 5 showed significant tumor regression and were classified as anti-PD-1-sensitive based on Response Evaluation Criteria in Solid Tumours (RECIST) criteria, while the other 5 exhibited no significant regression and were classified as anti-PD-1-resistant. To ensure comparability, the two cohorts were matched on age, sex, and therapeutic regimen. Notably, the anti-PD-1-resistant group displayed substantially higher pan-Lysine lactylation levels than the anti-PD-1-sensitive group (Fig. 1B), indicating an association between enhanced lysine lactylation and poor response to PD-1 blockade.

To analyze differences in cellular landscape between anti-PD-1-resistant and anti-PD-1-sensitive tumor tissues, we analyzed the publicly available single-cell RNA-sequencing dataset GSE207422, which includes fresh tumor tissues from 15 stage IIIA NSCLC patients treated with neoadjuvant anti-PD-1 therapy. Patients undergoing single-cell RNA sequencing were categorized based on the RECIST. The sensitive group comprised patients who achieved a complete response (CR) or partial response (PR) following anti-PD-1 therapy. The resistant group comprised patients presenting with stable disease (SD) or progressive disease (PD). However, all patients in the resistant group were SD, as no samples with PD were included in this dataset. Following stringent quality control and doublet removal, transcriptomes from 92,330 high-quality cells (median 1,256 detected genes per cell) were retained for downstream analyses. Using canonical lineage markers together with SingleR-based automatic annotation, these clusters were assigned to 12 major cell lineages, including B cells, Cancer stem cells, CD4⁺ T cells, CD8⁺ T cells, DC, Epithelial cells, Fibroblasts, Macrophages, Mast cells, Monocytes, Neutrophils, and NK cell (Fig. 1C). Next, we analyzed the cellular landscape of the tumor samples (Fig. 1D) and compared the resistant and the sensitive groups (Fig. 1E–H). Consistent with previous reports, tumors from the sensitive group exhibited markedly higher proportions of CD8⁺ T cells and B cells, cell populations closely associated with favorable immunotherapy outcomes. In contrast, the resistant group showed reduced CD8⁺ T cell infiltration and a notable enrichment of macrophages (Fig. 1E, F), suggesting an immunosuppressive TME that may inhibit effective anti-tumor immune responses. Given the crucial role of TAMs in shaping the tumor immune microenvironment and modulating response to immune checkpoint blockade [19], and considering their critical oncogenic roles in NSCLC, we focused on the TAMs cell cluster (Fig. 1G, H).

### KAT2A^+^ TAMs exhibited increased infiltration in the TME of the anti-PD-1-resistant group

**Fig. 2:**
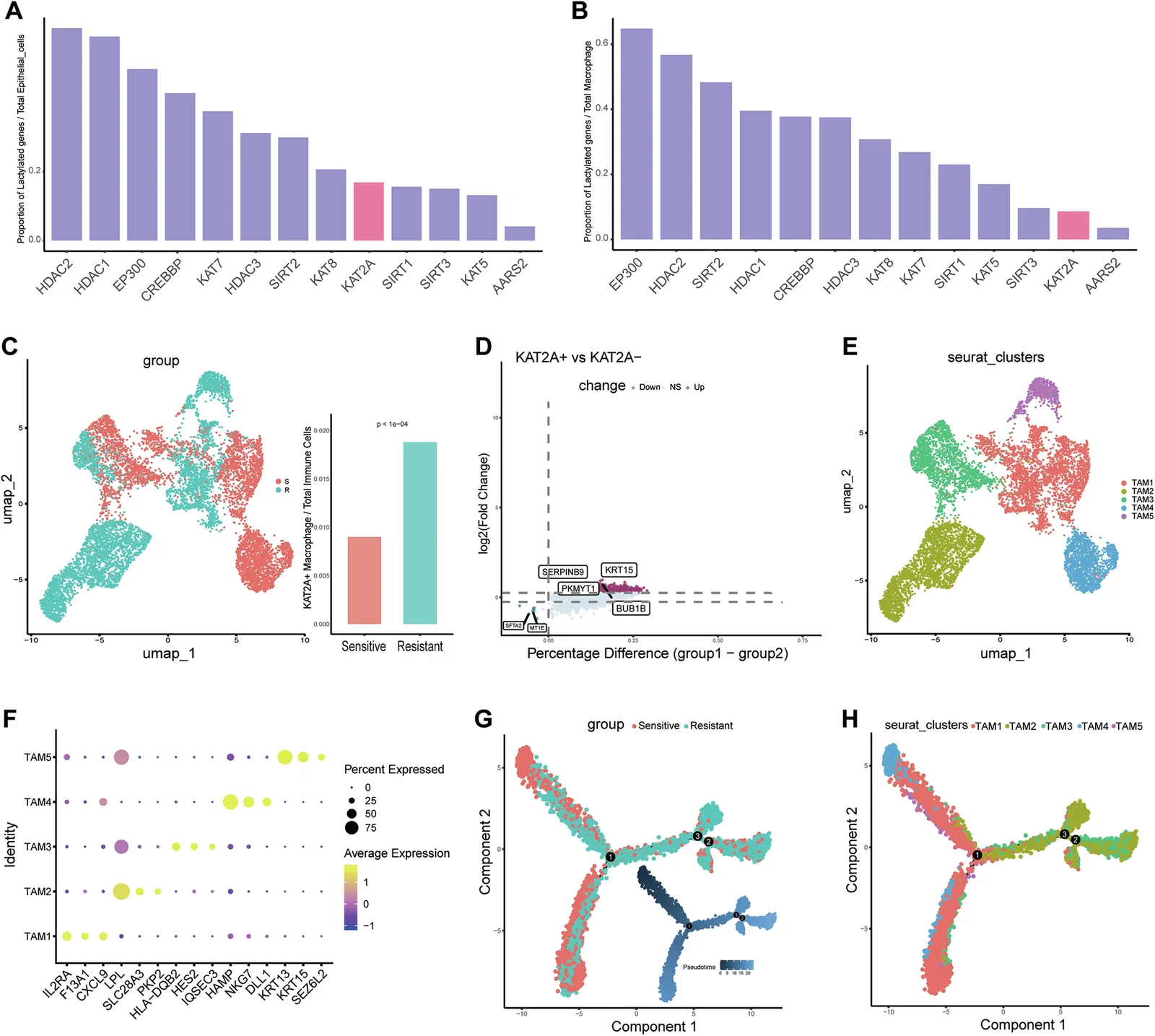
KAT2A^+^ TAMs exhibited increased infiltration in the anti-PD-1-resistant group. **A** Proportions of epithelial cells positive for each lactylationtransferase gene in the total epithelial cell population. **B** Proportions of macrophages positive for each lactylationtransferase gene in the total macrophage population. **C** The proportion of KAT2A^+^ TAMs among total immune cells was significantly higher in the anti-PD-1-resistant group compared to the sensitive group. The chi-square test was utilized to assess statistical differences. **D** Differential expression analysis, showing upregulated immunosuppressive phenotype-related genes in KAT2A^+^ TAMs. (KAT2A^+^ TAMs and KAT2A^-^ TAMs refer to TAMs expressing or not expressing KAT2A, respectively, as identified by single-cell RNA-sequencing analysis.). **E** UMAP visualization of the five subclusters of TAMs (TAM1-TAM5) identified by single-cell RNA-sequencing analysis. **F** Signature genes of the five TAM subclusters reveal immune suppressive properties in TAM2. **G**, **H** Pseudotime analysis using Monocle, indicating that TAM2 subset among the five TAM clusters (TAM1-TAM5) was preferentially located at the terminal end of the TAM differentiation trajectory and exhibited a notable alignment with the resistant group subcluster, suggesting a terminally differentiated state and properties related to immune suppression.

Lactylation modification impairs the function of immune cells within the TME, thereby facilitating tumor immune evasion. To investigate the role of lactyltransferases in anti-PD-1 therapy for LUAD, we evaluated the clinical prognosis of lactyltransferases family genes within the TCGA-LUAD cohort. Results showed that KAT2A demonstrated a certain predictive value for clinical prognosis, with an AUC of 0.612 (Figure S1A). Given the crucial roles of epithelial cells and TAMs in the TME, we therefore analyzed the expression of lactyltransferase family genes in these cell types using a single-cell dataset, focusing on the proportion of gene-positive cells. Analysis of scRNA-seq data showed that KAT2A ranked ninth in abundance among epithelial cells and eleventh in TAMs and was primarily expressed in the epithelial cells and macrophages of these LUAD cells (Fig. 2A, B), we focused our study on KAT2A. To determine the potential link between KAT2A and resistance to PD-1 blockade, we next utilized a Chi-square test to evaluate differences in the proportions of KAT2A^+^ TAMs among all immune cells (CD45^+^) between the anti-PD-1-sensitive and anti-PD-1-resistant groups. The results indicated that the proportion of KAT2A^+^ macrophages was significantly higher in the anti-PD-1-resistant group (Fig. 2C). Differential expression analysis further demonstrated that KAT2A^+^ TAMs exhibited markedly increased expression of genes linked to immunosuppressive functions including SERPINB9, PKMYT1, BUB1B, and KRT15 compared with KAT2A- TAMs [20–23] (Fig. 2D). We employed UMAP to visualize the distribution of all TAMs and performed unsupervised clustering, which revealed five transcriptionally defined macrophage subpopulations (TAM1-TAM5) (Fig. 2E). The TAM2 subpopulation was notably concentrated within the anti-PD-1-resistant group, as visualized by UMAP (Fig. 2C, E). The typical marker genes of TAM2 (LPL, SLC28A3, and PKP2) contribute to the immunosuppressive functions of TAMs [24, 25] (Fig. 2F). To analyse the dynamic transformation characteristics of different TAMs subtypes in the sensitive group and resistant group, we performed pseudotime analysis on 5 TAM subtypes via the monocle package. Finally, pseudotime analysis showed that TAM2 was preferentially located at the terminal end of the TAMs differentiation trajectory, and exhibited a notable alignment with the resistant group subcluster (Fig. 2G, H). In summary, our findings position TAMs as a key driver of resistance to anti-PD-1 therapy, potentially through directing the early differentiation trajectory and contributing to the immunosuppressive tumor microenvironment.

### KAT2A enhances resistance to anti-PD-1 therapy and reduces CD8^+^ T cells infiltration

**Fig. 3:**
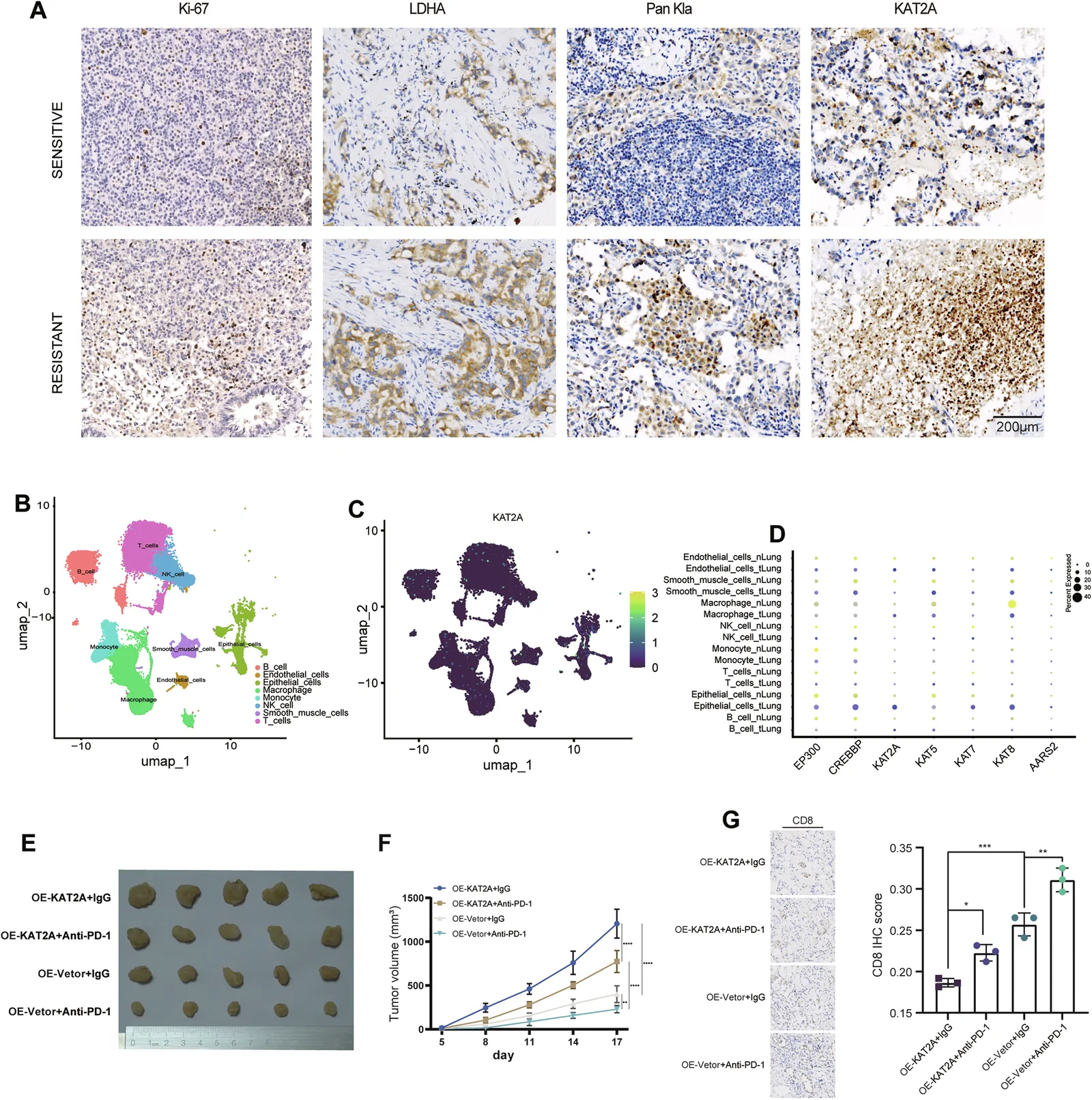
KAT2A modulates the tumor immune microenvironment and limits Anti-PD-1 efficacy in LUAD. **A** Representative immunohistochemical staining of paraffin-embedded patient tumor sections from anti-PD-1-sensitive and resistant patient groups for Ki-67, LDHA, Pan-Kla, and KAT2A. **B** Uniform Manifold Approximation and Projection (UMAP) visualization of cellular clusters in the validation dataset. **C** Distribution of KAT2A mRNA in different cell clusters, using the Nebulosa R-Package. **D** Percent expressed of lactylationtransferase genes in different cell clusters from normal and tumor tissue samples. **E** LUAD subcutaneous models were established using LA795 cells with KAT2A overexpression (OE-KAT2A) or control (OE-Vector). **F** Growth curves of subcutaneous LA795 tumors comparing KAT2A-overexpressing (OE-KAT2A) and control (OE-Vector) groups treated with either IgG or anti-PD-1 antibody (*n* = 5 biological replicates). **G** Representative IHC staining for CD8^+^ T cells in subcutaneous tumors with KAT2A-overexpressing or control that were treated with IgG or the anti-PD-1 antibody. CD8^+^ T cell infiltration was quantified for each group. Student’s *t* test was used for comparisons.

To investigate the potential regulatory role of lactylation in shaping gene expression and immune responsiveness under anti-PD-1 therapy, we first analyzed LUAD specimens from the cohort previously described, collected from patients who had received neoadjuvant therapy. Consistent with our previous results, the paraffin-embedded tumor sections demonstrated markedly elevated levels of tumor proliferative activity, glycolysis and lysine lactylation in the resistant group, accompanied by increased expression of the lactyltransferase KAT2A compared with the sensitive group (Fig. 3A). To determine whether this elevated lactylation observed in post-treatment resistant group was an intrinsic feature or therapy-induced, we analyzed pre-treatment biopsy specimens. Lactylation levels were assessed via immunohistochemistry (IHC) in tumor tissues obtained from percutaneous lung biopsies, bronchoscopy, or endobronchial ultrasound prior to the commencement of neoadjuvant therapy. Baseline analysis revealed no significant difference in lactylation levels between tumors that subsequently achieved sensitive group and those that progressed to resistant group (Figure S1B). These findings suggest that the heightened lactylation signature associated with treatment resistance is not a pre-existing trait but is instead adaptively upregulated in response to the selective pressure of neoadjuvant chemo-immunotherapy. These clinical data collectively suggested that enhanced lactylation and KAT2A expression may contribute to resistance to PD-1 blockade and correlate with poor survival in LUAD patients. We next validated the cellular distribution of KAT2A using the GSE131907 single-cell dataset, which comprises LUAD samples with well-defined cell annotations. The single cells were divided into eight cell clusters, as shown in Fig. 3B. KAT2A was primarily expressed in the epithelial cells and macrophages of these LUAD cells (Fig. 3C). We further analyzed the expression patterns of lysine lactyltransferase genes in LUAD and adjacent normal tissues. The results demonstrated that, compared to other genes in the set, KAT2A exhibited uniquely high expression positivity rates specifically within epithelial cells and macrophages of LUAD tissues (Fig. 3D). To investigate the role of KAT2A in anti-PD-1 therapy, we first performed an immune infiltration analysis using the TCGA-LUAD cohort. The ssGSEA results demonstrated that the KAT2A high expression exhibited reduced immune cell infiltration compared to the KAT2A low expression group (Figure S1C).

To evaluate the functional impact of KAT2A in vivo, we generated LA795 murine LUAD cells stably overexpressing KAT2A using lentiviral vectors (Shanghai Genomeditech). A total of 1 × 10⁶ LA795 cells carrying either empty vector or KAT2A-overexpression vector performed subcutaneous implantation immunocompetent T739 mice. Tumor volumes were measured every three days beginning on day 5. Anti-PD-1 antibody (#BE0146, BioXcell) was administered intraperitoneally every two days beginning on day 7. We sacrificed all mice on day 17 and dissected the subcutaneous tumors after carefully removing the surrounding hair (Fig. 3E, F). Subsequently, we processed a portion of the subcutaneous tumors into paraffin sections, and cryopreserved the remaining tissues for further research. The results of these in vivo experiments demonstrate that KAT2A suppresses CD8⁺ T cell infiltration (Fig. 3G), promotes LUAD progression, and induces resistance to anti-PD-1 therapy.

### KAT2A enhances recruitment of MDSCs into the LUAD microenvironment by promoting secretion of CXCL2

**Fig. 4:**
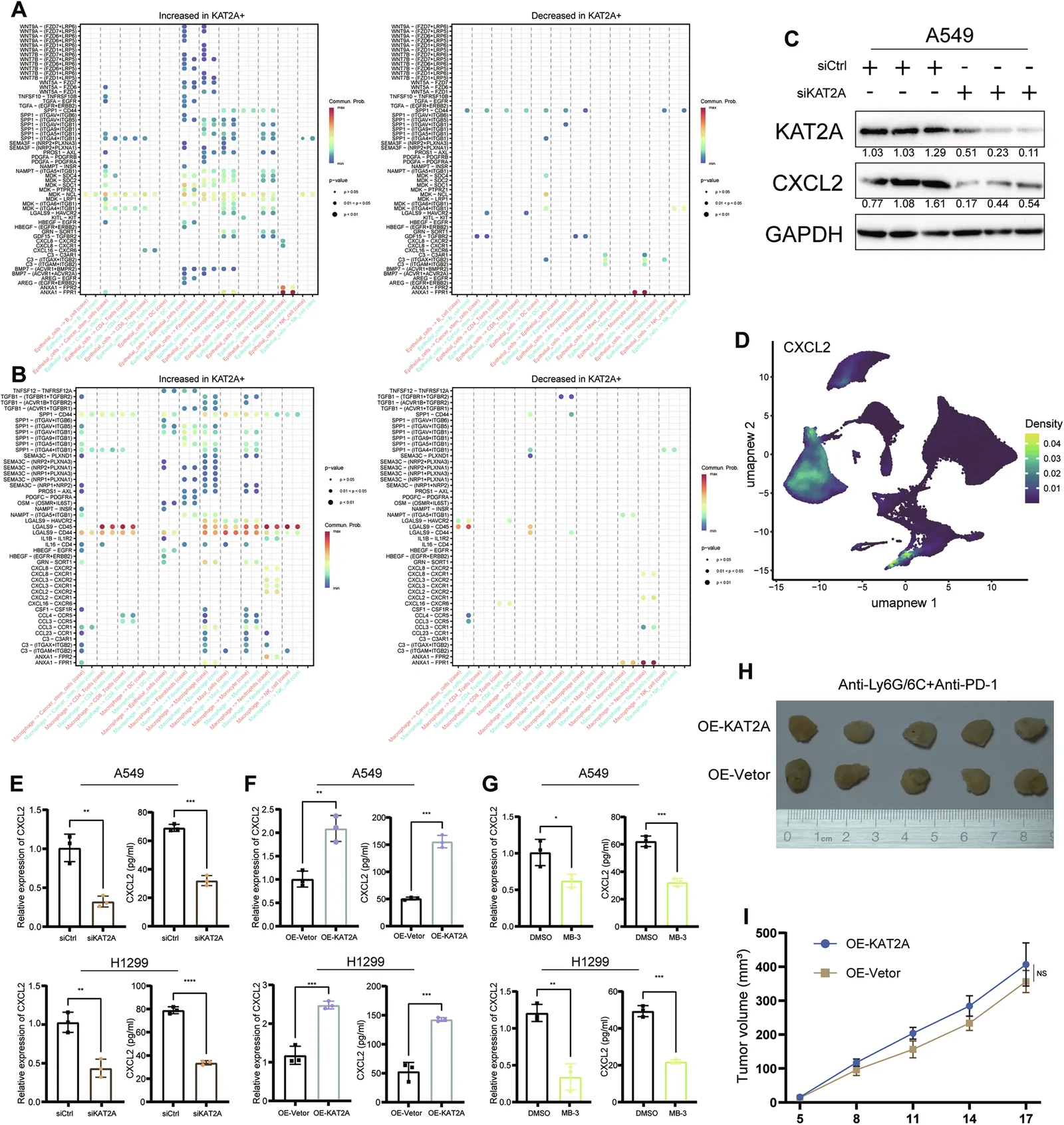
KAT2A increased recruitment of MDSCs into LUAD microenvironment by secretion of CXCL2. **A** Bubble plot showing the interaction patterns between KAT2A^+^ epithelial cells as signal senders and Recipient cells. **B** Bubble plot showing the interaction patterns between KAT2A^+^ Macrophages cells as signal senders and Recipient cells. Bubble size represents interaction strength, while color intensity indicates significance. **C** Western blot assay of KAT2A and CXCL2 expression in A549 cells transfected with siCtrl or siKAT2A. **D** Expression of CXCL2 across various cell clusters of LUAD in the validation dataset. Effect of KAT2A Knockdown (**E**) and Overexpression (**F**) on the expression and secretion of CXCL2 in A549 and H1299 Cells. **G** Effect of inhibition of KAT2A by MB-3 on the expression and secretion of CXCL2 in A549 and H1299 Cells. **H** In vivo experiments confirmed that the resistance of KAT2A to anti-PD-1 therapy primarily depended on MDSC recruitment (*n* = 5 biological replicates). The administration of anti-PD-1 was conducted as previously described. The anti-Ly6C/6 G antibody was administered by intraperitoneal injections at a dosage of 80.0 µg per mouse every other day (q.o.d.). **I** Tumor growth curves in T739 mice.

Within the TME, where cell-cell interactions play crucial roles in disease progression, we investigated potential interactions, with a focus on those between epithelial cells and macrophages with other cell types. The analysis revealed that KAT2A expression modulates intercellular communication mediated by CXC chemokine ligand-receptor pairs among epithelial cells, macrophages, and other cells within the microenvironment (Fig. 4A, B). Previous studies have demonstrated that CXCL16, CXCL8, CXCL3, and CXCL2 represent key inflammatory chemokines that efficiently recruit MDSCs, thereby fostering a protumor inflammatory milieu, supporting angiogenesis, and driving tumor progression [26]. Our results suggest that anti-PD-1 therapy resistance in LUAD may implicate the CXCL-CXCR signaling pathway, highlighting its potential role in immune evasion.

Subsequently, we focused on these four immune factors. KAT2A knockdown was found to markedly decrease only CXCL2 expression, as demonstrated by Western blotting (Fig. 4C). Analysis of single-cell RNA-sequencing data from anti-PD-1 treated samples revealed that CXCL2 expression was primarily localized to epithelial cells, macrophages, and monocytes (Fig.4D). Consistent with these findings, qPCR and ELISA assays performed on A549 and H1299 cell lines demonstrated that KAT2A knockdown significantly decreased CXCL2 mRNA levels and protein secretion, whereas KAT2A overexpression increased them (Fig. 4E, F). Furthermore, treatment with the selective KAT2A inhibitor MB-3 (10 μM for 48 h), substantially reduced CXCL2 transcription and secretion (Fig. 4G). This provides strong evidence that KAT2A positively regulates CXCL2 expression in LUAD cells.

By binding to C-X-C motif chemokine receptor 2 (CXCR2), their specific receptor, cytokines, such as CXCL2, promote the recruitment of MDSCs from the circulatory system into the tumor microenvironment [27]. The high infiltration of MDSCs in LUAD increases their resistance to anti-PD-1 therapy [28]. To investigate the mechanisms through which KAT2A promotes resistance to anti-PD-1 therapy in LUAD, we established animal models and administered an anti-Ly6C/6 G antibody (#BE0320, BioXcell, i.p. injection [80 μg/mouse, q.o.d.]) to deplete MDSCs in T739 mice. Subsequent experimental results demonstrated that KAT2A induced resistance to anti-PD-1 therapy was significantly attenuated after treatment with this antibody (Fig. 4H, I). These findings suggested that the role of KAT2A in mediating resistance to anti-PD-1 therapy largely depends on CXCL2-mediated recruitment of MDSCs.

### IGF2BP1 promotes CXCL2 accumulation via m⁶A modification on CXCL2 mRNA

**Fig. 5:**
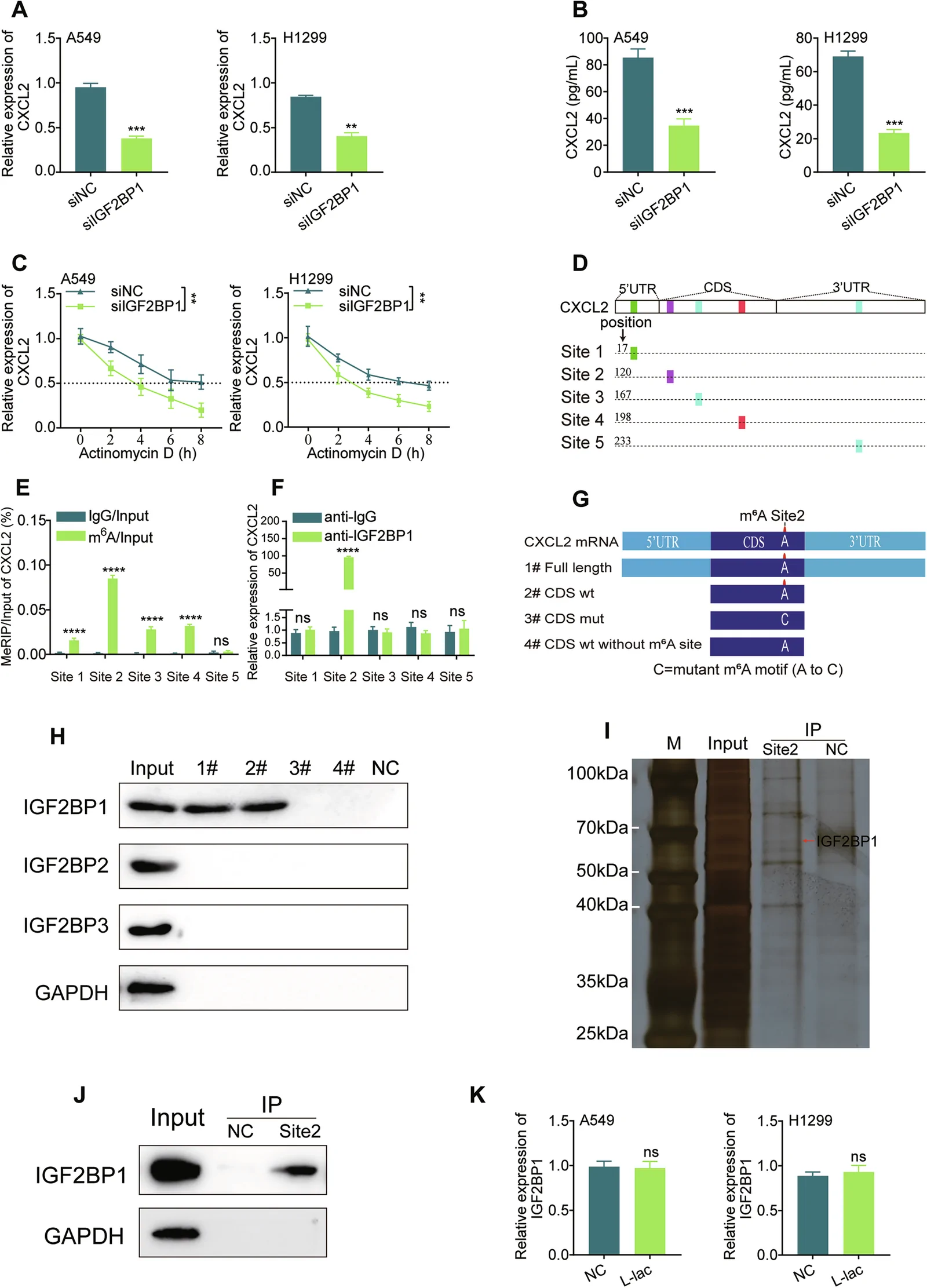
IGF2BP1 recognizes m⁶a-modified CXCL2 transcripts to enhance mRNA stability and promote secretion in LUAD cells. **A**, **B** The qPCR and ELISA analyses demonstrate reduced CXCL2 mRNA expression and protein secretion following IGF2BP1 knockdown in A549 and H1299 cells. **C** Actinomycin D chase assay showing decreased CXCL2 mRNA stability upon IGF2BP1 knockdown. **D** Schematic of predicted m⁶A methylation sites on CXCL2 mRNA. MeRIP-qPCR confirming m⁶A modification at multiple CXCL2 sites (**E**), and RIP-qPCR identifying IGF2BP1 binding specifically at site 2 (**F**). **G** Diagram of synthesized biotin-labeled CXCL2 RNA probes used for pulldown assays. **H** Western blot of pulldown products showing IGF2BP1 binding to full-length and wild-type CDS probes. Silver staining (**I**) and Western blot (**J**) verifying IGF2BP1 binding to the site-2 biotin probe. **K** qPCR analysis showing that lactate treatment (20 mM, 24 h) does not alter IGF2BP1 mRNA levels in A549 and H1299 cells.

Previous studies have reported that m⁶A modification of CXCL2 is a critical post-transcriptional regulatory mechanism. It modulates the mRNA stability, translation efficiency, and degradation rate of CXCL2, thus precisely controlling the expression of this key chemokine. Members of the IGF2BP family are m⁶A reader proteins that recognize m⁶A modifications and they selectively bind m⁶A-modified transcripts to enhance their stability. Accumulating evidence indicates their crucial involvement in regulating tumor microenvironmental remodeling. To elucidate the role of m⁶A modification in regulating CXCL2 expression, we knockdown IGF2BPs in A549 and H1299 LUAD cell lines. CXCL2 expression was then assessed using qPCR for mRNA and ELISA for protein. Knockdown of IGF2BP1 most significantly reduced both CXCL2 mRNA abundance and protein secretion in LUAD, as shown by the results (Fig. 5A, B). To further dissect the mechanism by which IGF2BP1 regulates CXCL2, we performed RNA stability assays using actinomycin D. IGF2BP1 knockdown significantly decreased the stability of CXCL2 mRNA (Fig. 5C). Since IGF2BP1 is a canonical m⁶A-binding protein, we hypothesized that it likely regulates the stability of CXCL2 in an m⁶A-dependent manner. Using the SRAMP database, we identified five putative m⁶A sites in the CXCL2 transcript, with three in the CDS, one in the 5′UTR, and one in the 3′UTR (Fig. 5D). To experimentally validate the accessibility of these predicted sites, we designed site-specific primers for all five candidate loci and performed MeRIP-qPCR. The results indicated that four sites (Site 1-4) are likely m⁶A modified (Fig. 5E). RIP assays further confirmed that IGF2BP1 specifically binds to Site 2 (Fig. 5F). Using RNA pull-down assays, we designed and synthesized four distinct biotin-labeled RNA probes to determine the direct binding sites of IGF2BP1 on CXCL2 mRNA (Fig. 5G). Subsequent immunoblot analysis revealed that only the full-length CXCL2 transcript and the CDS wt probe successfully pulled down IGF2BP1, but not IGF2BP2 or IGF2BP3 (Fig. 5H). Consistent results were observed using silver staining (Fig. 5I) and immunoblotting (Fig. 5J) following pull-down with a Site 2-specific probe, collectively confirming that IGF2BP1 directly and selectively recognizes the m⁶A-modified Site 2 within the CXCL2 CDS region to enhance CXCL2 mRNA stability.

To investigate whether lactate influences this regulatory axis by modulating IGF2BP1 expression, we treated A549 and H1299 cells with L-lactate (20 mM, 24 h). The results demonstrated that L-lactate treatment did not alter IGF2BP1 mRNA levels (Fig. 5K), suggesting that lactate does not affect the transcriptional regulation of IGF2BP1. Together, these results confirmed that IGF2BP1-mediated m⁶A modification on CXCL2 mRNA is critical for CXCL2 mRNA stability, whereas changes in lactate levels did not alter IGF2BP1 mRNA expression.

### Lactate upregulates CXCL2 expression through promoting the lactylation of IGF2BP1

**Fig. 6:**
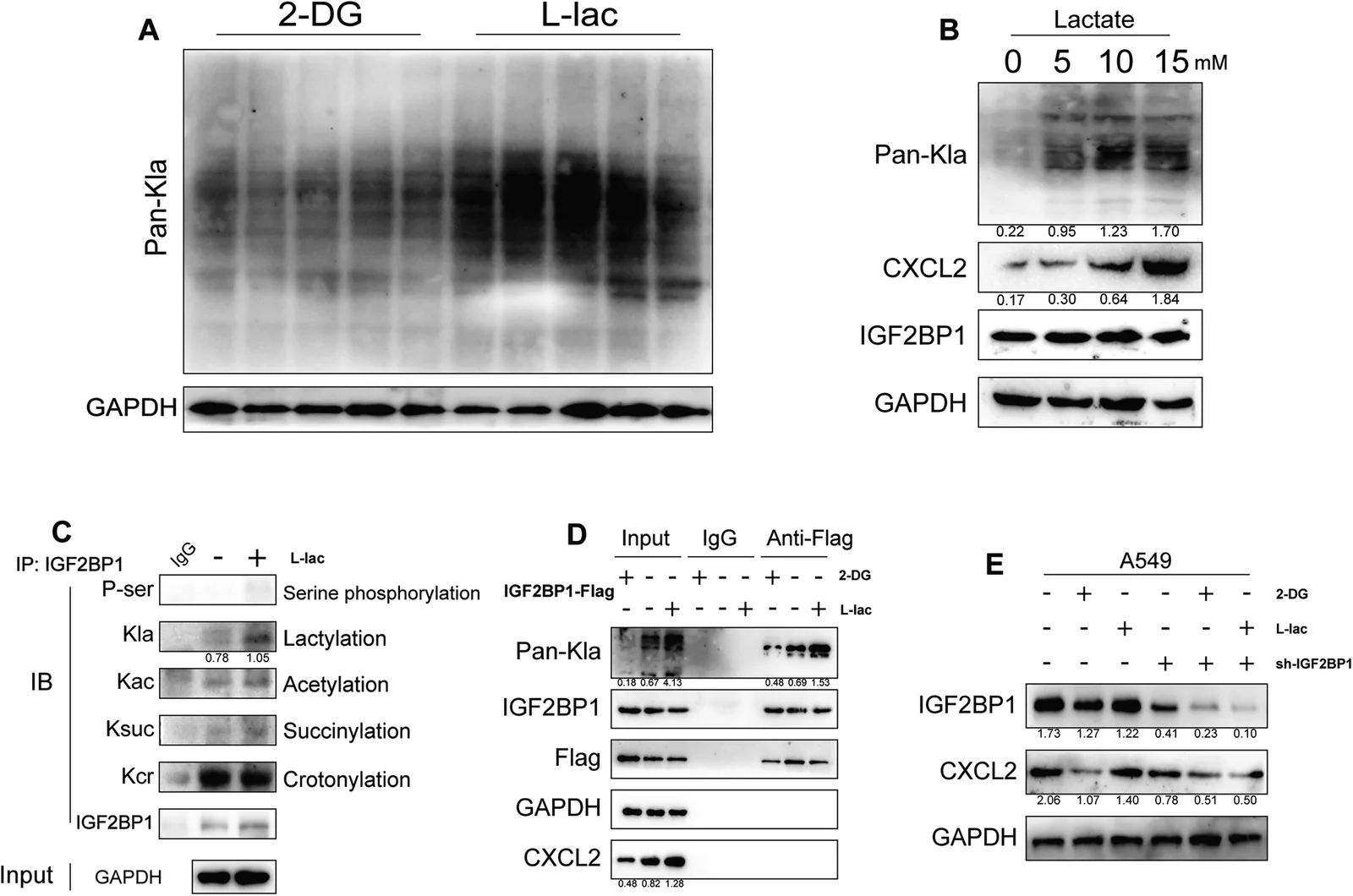
Lactate induces IGF2BP1 lactylation to modulate its function and promote CXCL2 expression. **A** A549 were exposed to 2-DG or the L-lactate for 24 h, and cell samples were collected for Western blot analysis of pan-Kla levels. **B** A549 cells were exposed to L-lactate (0, 5, 10 or 15 mM) for 24 h, and cell samples were collected for Western blot analysis of pan-Kla levels. **C** Immunoprecipitation and representative western blot analysis of IGF2BP1 post-translational modification after L-lactate treatment. Serine phosphorylation (P-ser), lactylation (Kla), acetylation (Kac), succinylation (Ksuc), and crotonylation (Kcr). **D** Immunoprecipitation (IP) using anti-Flag antibody-conjugated beads to pulldown exogenously overexpressed IGF2BP1-Flag in A549 cells, followed by Western blot analysis to detect IGF2BP1 lactylation in the 2-DG or L-lactate treatment groups. **E** Western blot analysis of the effect of altered lactate levels on CXCL2 expression in A549 cells transfected with empty vector or IGF2BP1-targeting shRNA.

It has been reported that m⁶A modification plays a crucial role in LUAD tumor immune evasion and immunotherapy responses, significantly influencing disease progression and treatment efficacy [29, 30]. Given that lactate serves as a substrate for lactylation, higher lactate levels lead to increased lactic acid availability, thereby enhancing protein lactylation. Based on preliminary experimental evidence, we hypothesize that KAT2A may regulate CXCL2 expression through lactylation modification. To test our hypothesis, we treated cells with 2-deoxy-D-glucose (2-DG; 10 mM, 24 h) to inhibit glycolysis and simulate a low-lactate environment, whereas a high-lactate condition was modeled using lactate (20 mM, 24 h). We found that treatment of A549 cells with 2-DG or L-lactate significantly altered the levels of pan-lysine lactylation (Fig. 6A). Increasing concentrations of L-lactate further enhanced cellular pan-lysine lactylation levels and concomitantly upregulated CXCL2 protein expression, whereas IGF2BP1 protein abundance remained unchanged (Fig. 6B). Clinical analysis of the TCGA-LUAD cohort demonstrated that IGF2BP1 exhibited time-dependent prognostic value, with significant predictive accuracy for 1-, 2-, and 3-year survival (Figure S1D). The Kaplan-Meier survival curves have shown that patients with high IGF2BP1 expression had significantly poorer overall survival (*P* = 0.012; Figure S1E), supporting a critical role of IGF2BP1 in LUAD progression.

Given that the aforementioned results indicate that lactate levels do not regulate the transcription and translation of IGF2BP1, to investigate whether alterations in lactate levels affect IGF2BP1 function through PTM, we systematically examined the PTM profile of IGF2BP1 by immunoprecipitation. The results showed that alterations in lactate levels specifically affected the lactylation of IGF2BP1, whereas the levels of its Serine phosphorylation (P-ser), acetylation (Kac), succinylation (Ksuc), and crotonylation (Kcr) showed no significant changes (Fig. 6C). To investigate the regulation of IGF2BP1 by lactate, we performed immunoprecipitation (IP) of IGF2BP1-Flag and found that L-lactate increased IGF2BP1 lactylation, while 2-DG diminished it. These changes in lactylation led to corresponding alterations in CXCL2 expression, with L-lactate increasing and 2-DG decreasing its levels. Importantly, the total expression level of IGF2BP1 was not statistically altered by L-lactate or 2-DG treatments, confirming a specific effect on its PTM (Fig. 6D). To determine if the L-lactate induced elevation of CXCL2 is dependent on IGF2BP1 lactylation, we knocked down IGF2BP1 under conditions of 2-DG or L-lactate treatment and evaluated the consequent effects on CXCL2 levels. Western blot analysis revealed that IGF2BP1 knockdown significantly reversed the effect of L-lactate on CXCL2 expression (Fig. 6E). Collectively, these results demonstrate that elevated lactate levels induce IGF2BP1 lactylation, which augments its ability to regulate CXCL2 mRNA via m⁶A modification, ultimately enhancing CXCL2 mRNA stability and protein expression.

### Lactylation of IGF2BP1 by KAT2A drives anti-PD-1 resistance in LUAD through CXCL2 upregulation

**Fig. 7:**
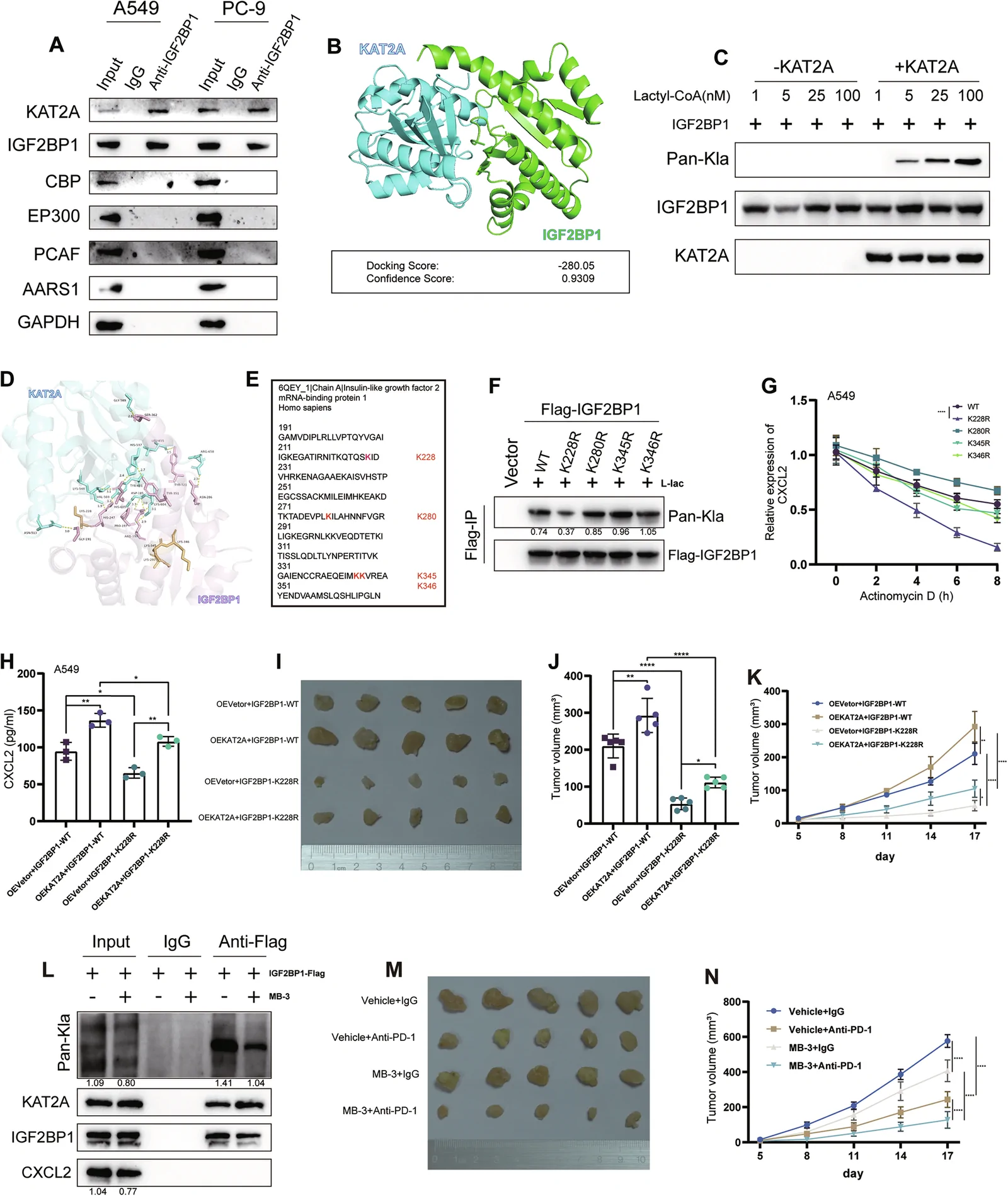
KAT2A mediated lactylation of IGF2BP1 confers resistance to anti-PD-1 therapy in LUAD. **A** IP followed by Western blot analysis to detect lactyltransferases that interact with IGF2BP1 in A549 and PC-9 cells. **B** Molecular docking analysis of the interaction between KAT2A and IGF2BP1 proteins using HDOCK. (C) KAT2A-mediated IGF2BP1 lactylation was analyzed by mixing IGF2BP1 and lactyl-CoA (1–100 nM) with or without the addition of purified KAT2A from 293 T cells. Immunoblotting with the indicated antibodies was performed. **D**, **E** Predicted potential KAT2A-mediated lactylation sites on IGF2BP1 (K228, K280, K345, K346). **F** A549 cells transfected with the indicated IGF2BP1 site mutations were treated with L-lactate, followed by immunoprecipitation and Western blotting for lactylation of IGF2BP1. **G** Actinomycin D chase assay demonstrates decreased CXCL2 mRNA stability following the IGF2BP1-K228 mutation. **H** The IGF2BP1-K228R mutation attenuates the upregulation of CXCL2 protein levels induced by KAT2A overexpression in A549 cells. **I**, **J** The IGF2BP1-K228R mutation partially counteracts the promoting effect of KAT2A overexpression on tumor volume in a subcutaneous tumor model. **K** The IGF2BP1-K228R mutation partially reverses the pro-tumorigenic effect of KAT2A overexpression on tumor growth rate. **L** A549 cells treated with or without the KAT2A inhibitor MB-3 were subjected to IP followed by Western blot analysis to detect IGF2BP1 lactylation. **M**, **N** Subcutaneous tumor growth in mice treated with or without anti-KAT2A therapy (MB-3) in combination with anti-PD-1 therapy. Inhibition of KAT2A was achieved using MB-3, which was administered via intraperitoneal (i.p.) injections daily starting on Day 7 (*n* = 5 biological replicates).

The above data confirmed that lactylation modification of IGF2BP1 modulates CXCL2 expression, although the specific lactyltransferases responsible for this modification in LUAD cells remain unclear. To verify the universality of IGF2BP1 lactylation modification in LUAD cell lines, we initially performed co-immunoprecipitation in the new cell line PC-9 using pan-lysine lactylation antibody-conjugated magnetic beads on cell lysates to comprehensively capture lactylated proteins. Subsequent Western Blot analysis of the precipitated products using an anti-IGF2BP1 antibody clearly detected IGF2BP1 bands, demonstrating that lactylation modification was also present on IGF2BP1 under high-lactate conditions in PC-9 cells (Figure S1F). To further elucidate the regulatory mechanism of IGF2BP1 lactylation in LUAD, we employed immunoprecipitation to investigate the protein-protein interactions between IGF2BP1 and several commonly studied lactyltransferases. Our results demonstrated that IGF2BP1 specifically interacted with KAT2A, whereas no interactions were detected with other lactyltransferases, including CBP, EP300, PCAF, and AARS1 (Fig. 7A). These findings suggest that KAT2A may function as the lactyltransferase responsible for catalyzing the lactylation of IGF2BP1. We therefore examined the potential interaction between KAT2A and members of the IGF2BP family. Using the template-based molecular docking server HDOCK, we generated several predicted complex conformations between KAT2A (as the ligand) and IGF2BPs (as receptors) (Fig. 7B and Figure S1G, H). The model exhibiting the most favorable docking free energy (−280.05 kcal/mol) and highest confidence score (0.9309) for the KAT2A-IGF2BP1 interaction was selected for subsequent analyses.

To investigate whether KAT2A directly lactylates IGF2BP1, we incubated purified KAT2A with purified WT IGF2BP1 in the presence of lactyl-CoA. Our data demonstrated that KAT2A dosage-dependently lactylated WT IGF2BP1 as detected by immunoblotting analyses. However, IGF2BP1 lactylation did not occur in the absence of KAT2A between 1–100 nM lactylCoA (Fig. 7C). These results suggested that IGF2BP1 lactylation may not occur in an enzyme-independent manner and requires the catalysis of a protein lactyltransferase. These results indicate that KAT2A is a bona fide lactyltransferase for IGF2BP1. Our results further corroborate that KAT2A may mediate the lactylation of IGF2BP1 through direct protein-protein interaction, consequently modulating its biological activity. To further delineate potential lactylation sites on IGF2BP1, we performed in-depth structural modeling analysis using PyMOL based on the IGF2BP1 interaction complex. We analyzed and predicted several candidate lysine as putative lactylation sites, with K228, K280, K345, and K346 identified as the most likely modified lysine (Fig. 7D, E). To identify the key lactylation site of IGF2BP1, we generated lactylation-defective mutants (K to R) at these four candidate sites. We found that only the IGF2BP1-K228R mutant showed reduced lactylation upon L-lactate treatment (Fig. 7F). These results demonstrate that IGF2BP1 is lactylated at K228 upon lactate treatment. Next, we investigated the functional impact of K228 lactylation on IGF2BP1. To determine whether IGF2BP1-K228 lactylation enhances its binding affinity for m⁶A sites, we assessed the stability of CXCL2 mRNA using actinomycin D and quantified CXCL2 protein levels using ELISA. These results indicate that IGF2BP1-K228 lactylation enhances CXCL2 mRNA stability and upregulates its protein expression in an m⁶A-dependent manner (Fig. 7G and Figure S1I).

To demonstrate that KAT2A mediates lactate-driven downstream signaling by lactylating IGF2BP1 at K228, an event that upregulates CXCL2 expression and enhances resistance to anti-PD-1 therapy, we conducted a rescue experiment. We established four experimental groups: OEVector+IGF2BP1-WT, OEKAT2A+IGF2BP1-WT, OEVector+IGF2BP1-K228R, and OEKAT2A+IGF2BP1-K228R. Consistently, the IGF2BP1-K228R mutation attenuated KAT2A overexpression-induced upregulation of CXCL2 protein levels in A549 cells (Fig. 7H). Similarly, in the subcutaneous tumor model, the IGF2BP1-K228R mutation partially counteracted the pro-tumorigenic effects of KAT2A overexpression on tumor volume (Fig. 7I, J) and growth rate (Fig. 7K). These findings suggest the lactylation modification of IGF2BP1 at K228, mediated by KAT2A, is a necessary pathway for lactate to drive downstream signals. Furthermore, under lactate conditions, treatment with the KAT2A inhibitor MB-3 reduced the lactylation level of IGF2BP1 (Fig. 7L), thereby confirming that KAT2A is a key writer for the lactylation modification of IGF2BP1. Based on our previous investigations, we employed MB-3 to inhibit KAT2A in vivo. The results demonstrated that KAT2A suppression significantly reduced tumor volume, delayed tumor growth, and enhanced the sensitivity of LUAD to anti-PD-1 therapy (Fig. 7M, N). These findings suggest that targeting KAT2A may serve as an effective strategy to potentiate the sensitivity of LUAD to anti-PD-1 treatment.

## Discussion

Protein lactylation plays a pivotal role in regulating tumor progression, reshaping the immune microenvironment, and facilitating the formation of an immunosuppressive tumor microenvironment [31–35]. In this study, we demonstrated that in PD-1-resistant lung adenocarcinoma, KAT2A catalyzes lactylation at the K228 site of IGF2BP1 within a high-lactate microenvironment. Functioning as a lactyltransferase, KAT2A mediates the lactylation of IGF2BP1, which enhances its specific recognition of m⁶A-modified CXCL2 mRNA, thereby promoting CXCL2 mRNA stability and translation. The KAT2A–IGF2BP1–CXCL2 axis drives resistance to anti-PD-1 therapy by recruiting MDSCs and reducing CD8⁺ T-cell infiltration, ultimately accelerating tumor progression. Furthermore, we provide evidence that combined treatment with the KAT2A inhibitor MB-3 and an anti-PD-1 antibody represents a promising therapeutic strategy to effectively counteract tumor immune evasion and growth during immunotherapy.

In the tumor immune microenvironment, lactylation interacts with various immunosuppressive cells through the remodeling of the tumor immunosuppressive microenvironment (TIME). For example, histone lactylation can stimulate transcriptional activation of tumor-promoting genes within chromatin, such as immune checkpoint molecules and epithelial-mesenchymal transition (EMT) factors, resulting in attenuation of antitumor immunity and ultimately leading to immune therapy resistance [34]. Furthermore, lactylation remodels the TME by modulating the functionality of diverse immune cell populations. Collectively, these alterations drive tumor resistance to therapies such as immune checkpoint inhibitors [35]. Thus, the identification of patients who are likely to benefit from immunotherapy and the development of personalized combinatorial regimens represent major hurdles to the successful implementation of immunotherapy for LUAD and other malignancies.

Although KAT2A (GCN5) is classically recognized for its histone acetyltransferase activity, emerging evidence suggests that acyltransferases may exhibit broader substrate selectivity, including involvement in lactylation and other potential protein modifications. For instance, in hepatocellular carcinoma, as a lysine acetyltransferase, KAT2A interacts with ALDOB to suppress H3K9 acetylation at the TGFB1 promoter, leading to reduced TGF-β expression. This process contributes to impaired CD8^+^ T cell infiltration and function, ultimately promoting immune evasion [36]. Genetic or pharmacological inhibition of KAT2A suppresses proliferation and induces differentiation, highlighting its potential as a therapeutic target in this subset. KAT2A also functions beyond canonical acetylation. It partners with ACSS2 to act as a lactyltransferase, facilitating histone H3 lactylation and driving expression of oncogenic pathways such as Wnt/β-catenin, NF-κB, and PD-L1 [37]. This mechanism promotes brain tumor growth and immune evasion, and disruption of the ACSS2–KAT2A axis enhances the efficacy of anti-PD-1 therapy. Additionally, KAT2A-mediated non-histone modifications contribute to tumor progression. In breast cancer, KAT2A catalyzes RCC2 lactylation, which stabilizes MAD2L1 mRNA and accelerates proliferation under high-glucose conditions. Targeting RCC2 lactylation effectively curbs tumor growth [38, 39]. In contrast, our study unveils a unique axis in which KAT2A catalyzes the lactylation of the epitranscriptomic reader IGF2BP1, leading to the upregulation of CXCL2 expression, recruitment of MDSCs, and consequent immunotherapy resistance. This suggests that the KAT2A-mediated lactylation pathway may be operational across various cancer types, but the specific downstream effectors and pathological outcomes it drives are likely context-dependent.

Dysregulation of IGF2BP1 is closely associated with the progression of various malignancies, including acute myeloid leukemia, breast cancer and lung adenocarcinoma [40–42]. Therefore, these findings underscore IGF2BP1 as a critical m⁶A reader that post-transcriptionally regulates key oncogenic pathways, offering a promising diagnostic biomarker and therapeutic target across diverse cancers. Our findings demonstrate that IGF2BP1 significantly upregulates CXCL2 expression through m⁶A modification. Based on these findings, further mechanistic exploration reveals that IGF2BP1’s ability of m⁶A-modifying is modulated via lactylation-mediated post-translational modification, and this modulation can reinforce its ability to modify m⁶A of RNAs. Our discovery that IGF2BP1 is regulated by lactylation suggests the compelling hypothesis that other m⁶A readers (e.g., YTHDF1/2/3, HNRNP family proteins) may also undergo similar lactylation, especially in metabolic or inflammatory tumor microenvironments where lactate levels are frequently elevated. This potential broader regulatory network warrants further investigation to elucidate the interplay between epitranscriptomic control and metabolic reprogramming in cancer immunity.

In the TME of LUAD, MDSCs have been identified as key mediators of immunosuppression and resistance to immune therapy [27, 43, 44]. Recent studies demonstrate that MDSCs contribute to tumor progression by suppressing anti-tumor immune responses, particularly through mechanisms involving metabolic alterations and cytokine signaling [45]. For instance, FFAR2-expressing MDSCs enhance immunosuppression via the Gαq/calcium/PPAR-γ axis, upregulating Arg1 expression and depleting L-arginine, which leads to CD8^+^ T cell dysfunction and immune evasion [27]. Additionally, protein kinase Ci signaling promotes YAP1-dependent expression of CXCL5, recruiting MDSCs to the tumor site and fostering resistance to anti-PD-1 therapy by reducing CD8^+^ T cell infiltration [43]. In the context of metastasis, MDSCs with high PD-L1 expression are crucial for forming the premetastatic niche, creating an immunosuppressive environment that facilitates LUAD progression [46]. Our experimental results showed that depletion of MDSCs in T739 mouse models significantly attenuated KAT2A-induced anti-PD-1 therapy resistance. These findings underscore the pivotal role of MDSCs in driving immunotherapy resistance in LUAD and highlight the potential of therapeutic strategies aimed at MDSCs inhibition to improve clinical outcomes [47].

Our integrated analysis, combining single-cell data with experimental validation, reveals that KAT2A drives resistance to anti-PD-1 therapy in LUAD through a defined molecular cascade. Mechanistically, KAT2A facilitates the lactylation of the m⁶A reader IGF2BP1, thereby enhancing its RNA-binding and stabilizing activity. This lactylated IGF2BP1, in turn, specifically binds to and stabilizes CXCL2 mRNA in an m⁶A-dependent manner, leading to increased CXCL2 secretion. The elevated CXCL2 chemokine then recruits MDSCs into the tumor microenvironment, which ultimately suppresses CD8⁺ T cell infiltration and fosters immunotherapy resistance. Crucially, our in vivo assays demonstrate that pharmacological inhibition of KAT2A with MB-3 sensitizes LUAD to anti-PD-1 therapy. Collectively, these findings identify KAT2A as a key mediator of immune resistance and a candidate target for combination therapy. The therapeutic potential and safety profile of targeting the KAT2A-IGF2BP1-CXCL2 axis in conjunction with immune checkpoint blockade warrant further preclinical and clinical investigation. This mechanistic insight offers a rationale for adjunctive therapeutic strategies aimed at reducing immunosuppressive components within the TME, thereby restoring a positive immunoregulatory feedback loop and potentially improving responsiveness to immunotherapy.

The study limitations due to the cohort size, constrained by the stringent requirement for paired, high-quality pre- and post-treatment tumor specimens from patients who completed neoadjuvant chemo-immunotherapy, which may limit generalizability, necessitating larger-scale, multi-center validation. Additionally, the analyzed single-cell dataset lacked samples from PD patients, with the resistant cohort comprising only SD cases, thus future investigations should incorporate PD samples to provide more direct evidence on therapeutic resistance mechanisms. Considering the broad roles of KAT2A and IGF2BP1 in normal cell metabolism and gene regulation, future efforts must focus on developing targeted strategies to maximize therapeutic efficacy against tumors while reducing disruption of essential cellular processes in normal cells.

## Methods

### Data acquisition and quality control

Single-cell RNA-sequencing data analyzed in this study were acquired from two GEO datasets. The GSE207422 dataset comprises 15 NSCLC samples following PD-1 blockade therapy, encompassing 24,292 genes and 91,840 cells. Additionally, the GSE131907 dataset contains scRNA-seq profiles of 208,506 single cells derived from 58 samples obtained from 44 patients pathologically diagnosed with LUAD. These samples consist of 11 primary tumors, 11 distant normal lung tissues, 10 normal lymph nodes, and 10 brain metastases. The quality control criteria were as follows: nFeatureRNA was between 300 and 7500, mitochondrial gene expression percentage (percent_mito) was less than 20%, and each gene was expressed in at least three cells.

To identify and eliminate potential doublets, we employed DoubletFinder v2.0.3. The expected number of doublets was calculated based on an assumed doublet rate of 7.5–8%, following 10X Genomics guidelines, and using the formula: nExp_poi = round (0.08 × N × N/10000), where N is the number of cells in the sample. For doublet prediction, we used 20 principal components (PCs = 1:20) and the following parameters: pN = 0.25, pK = 0.09, nExp = nExp_poi, reuse.pANN = FALSE, sct = FALSE. These settings were based on the recommended defaults in the official DoubletFinder tutorial.

Transcriptome profiling data with clinical information were obtained from the TCGA-LUAD project by R (version 4.4.1) with the R package TCGAbiolinks.

### Data integration, dimensionality reduction, and clustering

We performed data integration using the IntegrateLayers function from Seurat 5 with the method parameter set to CCAIntegration to correct for batch effects during integration. After integration, principal component analysis (PCA) was conducted on the integrated dataset, which was then embedded into a low-dimensional space using Uniform Manifold Approximation and Projection (UMAP) based on a selected number of principal components. Clusters were generated by a graph-based method using the FindClusters function from the Seurat package (v5.1.0). They were assigned to cell types by consulting the expression of known marker genes and by using automated annotation from the SingleR package (2.8.0).

### Cell-type identification

In order to determine cell types, we conducted differential expression analysis across clusters using the FindAllMarkers() function in Seurat. Marker genes were defined as those with an adjusted *p* value < 0.05, expression in more than 25% of cells within the cluster (min.pct = 0.25), and log_2_FoldChange > 0.25. For each cluster, the top differentially expressed genes were considered cluster-specific highly expressed genes. To determine the most probable cell-type identity for each cluster, we compared these cluster-specific markers with curated reference databases, such as CellMarker. Annotation was carried out manually by examining the expression patterns of canonical lineage markers and established cell-type-specific genes.

The SingleR package was used to support and cross-validate our manual annotations. This package employs reference transcriptomic datasets to determine cell identities. We used the results from SingleR as a secondary reference and compared them to our primary marker-based annotation strategy. Furthermore, we calculated Spearman correlation coefficients between the average expression profiles of all clusters to evaluate transcriptional similarity. Clusters with highly consistent expression patterns and overlapping marker gene expression were considered for combining subtypes in order to prevent artificial over-segmentation. The final cell-type labels were established by combining data from marker gene analysis, database matching, SingleR prediction, and inter-cluster correlation.

### Pseudotime trajectory analysis

Pseudotime trajectories were constructed using the Monocle 2 algorithm. This algorithm reduces highdimensional gene expression profiles into a low-dimensional space and arranges the cells into trajectories with branching points. Dynamic expression heatmaps were constructed using the plot_pseudotime_heatmap function.

### Cell communication analysis

Cell-cell communication networks within the tumor microenvironment were inferred using the CellChat v1.1.3R package based on receptor-ligand interactions. The probability of communication—measuring the likelihood of ligand-receptor interactions—and the number of interactions were calculated to construct these networks. Interactions between any two cell populations were visualized, and scatter plots were generated to display the major signaling senders, or signal sources, and receivers, or targets, in a two-dimensional space; this visualization helped identify the main contributors of outgoing and incoming signals, particularly among immune cell types. Subsequently, a pattern recognition method was applied to elucidate how multiple immune cell types and signaling pathways coordinate their interactions within the tumor microenvironment.

### Immune infiltration evaluation

To evaluate the immune cell infiltration levels in TCGA-LUAD samples, we employed single-sample gene set enrichment analysis (ssGSEA) through the GSVA R package. This approach quantified the relative abundance of 28 distinct immune cell populations by analyzing the expression patterns of published immune cell-specific gene signatures [48]. Subsequently, we performed comparative analyses between subgroups stratified by target gene expression levels.

### Maintenance and transfection of LUAD cell lines

The human LUAD cell lines A549, H1299 (passages 25–35) and PC-9 (passages 20–30) were obtained from Chinese Academy of Sciences, Shanghai, China, authenticated by STR profiling, and tested negative for mycoplasma contamination. Cells were used within 50 cumulative passages from the original stock to ensure phenotypic stability. All cells were maintained in DMEM supplemented with 10% FBS (Gibco, USA, A5670701) and 1% penicillin-streptomycin (Fuheng biology, Shanghai, K002TM) at 37 °C in a 5% CO2 atmosphere. For gene manipulation, cells were transfected with plasmids designed for either overexpression or short hairpin RNA (shRNA)-mediated knockdown of target genes using Lipofectamine 3000 (Gibco, USA, L3000015).

### In vivo assays

All experimental mice were bred and maintained under specific pathogen-free conditions at the Laboratory Animal Center, Shanghai Tongji Hospital. In vivo models were established using the mouse LUAD cell line (LA795 cells) in female immunocompetent T739 mice. Following stable transfection of LA795 cells with KAT2A using a viral vector solution prepared by Shanghai Genomeditech Co., Ltd., the cells were used to establish subcutaneous tumor models. Mice were monitored daily for tumor formation and overall health. The drugs (such as the Anti-PD-1 agent and MB-3) and their corresponding control agents were randomly administered to mice via intraperitoneal injection (*n* = 5 per group). Tumor diameters—length (*L*) and width (*W*)—were measured using calipers, and tumor volume was calculated using the formula: 0.5 × *L* × *W*². All mice were sacrificed on day 17 or when the tumor volume reached 2000 mm³. Tumors were surgically excised, weighed, and subsequently divided into portions for either fixation in 10% neutral-buffered formalin (for 24 h) for histological examination or snap-freezing in liquid nitrogen for subsequent molecular analyses. Mice were euthanized via intraperitoneal injection of sodium pentobarbital (300 mg/kg) in strict accordance with institutional animal ethics guidelines and the manufacturer’s recommendations. Randomization and blinding were not employed in this study.

### Clinical samples collection

Small pieces (0.3–0.6 cm^3^) of lung cancer tissues and adjacent non-tumorous tissues were taken from surgically resected lung specimens as part of the lung cancer biobanking process at the Tongji Hospital of Tongji University (Shanghai, China) with patients’ informed consent. The research protocol was approved by the Ethics Committee of the Tongji Hospital of Tongji University. The entire experimental protocol was conducted in compliance with the guidelines. Samples were confirmed as tumor or normal tissue on the basis of histopathological assessment. The diagnosis of each case was confirmed by pathologists at Tongji Hospital of Tongji University.

### qPCR analysis

Samples of tumor were analyzed for levels of CXCL2, IGF2BP1 and GAPDH mRNA by RT-qPCR, and levels of m⁶A at specific sites were quantified by methylated RNA immunoprecipitation followed by qPCR (MeRIP-qPCR). For RT-qPCR, total RNA was extracted using an RNA extraction kit (Sikejie, China, AC0202) and reverse-transcribed using a cDNA synthesis kit (Sikejie, China, AG0305). qPCR reactions were conducted with mix and gene-specific primers on a StepOnePlus Real-Time PCR System. Relative mRNA levels were calculated using the ΔΔCt method and normalized to the expression of GAPDH. The sequences of primers used for RT-qPCR are listed in Supplementary Table 1.

### Co-immunoprecipitation, Western blotting and immunohistochemistry

Co-IP assays were performed using Classic Magnetic Protein A/ G IP Kits (Epizyme, Shanghai, China). Briefly, protein lysates were incubated with antibodies for 1 h, followed by incubation with pre-washed magnetic beads for an additional hour. After magnetic separation for 1 min, the bead-antibody-protein complexes were washed four times with lysis buffer to remove non-specific interactions. Finally, the immunoprecipitated proteins were separated by SDS-PAGE for downstream analysis. For western blot analysis, protein lysates from LUAD cell lines were prepared using RIPA buffer supplemented with protease and phosphatase inhibitors, and proteins were then separated by SDS-PAGE and transferred to 0.22 μm PVDF membranes. The membranes were blocked with 5% skim milk in 37 °C and then incubated overnight at 4 °C with primary antibodies against human pan-lysine lactylation (ABclonal, China, A23004), KAT2A (CST, USA, #3305), CXCL2 (CST, USA, #24376), IGF2BP1 (Proteintech, China, 22803-1-AP), IGF2BP2 (Proteintech, China, 11601-1-AP), IGF2BP3 (Proteintech, China, 14642-1-AP) FLAG (Proteintech, China, 80801-2-RR), CBP (Proteintech, China, 22277-1-AP), EP300 (Proteintech, China, 20695-1-AP), and GAPDH (Abcam, UK, ab181602). After washing, the membranes were incubated with horseradish peroxidase (HRP)-conjugated secondary antibodies (Abcam, UK, ab205718) at 37 °C for 1 h. Protein bands were visualized using an enhanced chemiluminescence detection kit (Yamei, China) and quantified by densitometry using a Windows system with ImageJ software.

IHC was performed on formalin-fixed paraffin-embedded tissue sections. After deparaffinization and rehydration, tissue sections were incubated overnight at 4 °C with primary antibodies against Ki67, LDHA, Pan-Kla, KAT2A or CD8 and then washed and incubated with HRP-conjugated secondary antibodies. Color development was achieved using diaminobenzidine chromogen, and the sections were counterstained with hematoxylin. Sections were visualized, and images were captured using a light microscope. The intensity of staining was quantified using ImageJ software to assess relative protein expression levels.

### Actinomycin D and cycloheximide assay

To assess mRNA stability, cells were treated with 5 µg/mL actinomycin D (Selleck, USA, S8964) to inhibit transcription, and RNA was extracted before and at 0, 2, 4, 6, and 8 h post-treatment. CXCL2 mRNA was quantified by RT-qPCR, and the levels were normalized to time zero to determine the mRNA decay rate.

### RNA-binding protein immunoprecipitation (RIP) assay

RIP was used to identify RNA-binding proteins that interact with CXCL2 mRNA. A549 cell lysates were incubated with specific anti-IGF2BP1, IGF2BP2 or IGF2BP3 antibodies, and immune complexes were collected using magnetic protein G beads. RNA bound to the antibodies was extracted and analyzed by qPCR to quantify CXCL2 mRNA [49].

### RNA-pulldown assay

For MeRIP-qPCR, the MeRIP assay was conducted using a m⁶A MeRIP Kit (GenSeq, China, GS-ET-001A) following the manufacturer’s instructions with slight modifications. Briefly, total RNA was extracted and its quality was assessed using 1% agarose gel electrophoresis. RNA samples (>100 µg) were fragmented into ~200 nt using the kit’s fragmentation buffer at 70 °C for 5 min. Then, the fragmented RNA was immunoprecipitated with m⁶A-specific antibodies coupled to magnetic beads. After a series of washes with low-and high-salt buffers to remove non-specific bindings, the RNA-antibody-bead complexes were eluted. Subsequently, the RNA was purified using the kit’s purification protocol involving magnetic beads and buffers. Finally, the purified RNA was eluted and quantified for downstream applications such as qPCR or sequencing. The kit also included control IgG antibodies for mock IP experiments to assess background noise. The entire process was carried out using nuclease-free materials and reagents to ensure the integrity of the RNA samples.

Biotinylated RNA probes corresponding to m⁶A-modified sites in CXCL2 were synthesized and incubated with cytoplasmic lysates, and RNA complexes were then collected using streptavidin beads. Proteins bound to RNA probes were eluted, separated by SDS-PAGE, and analyzed by western blotting as described above.

### In vitro IGF2BP1 lactylation assay

To analyze KAT2A-catalyzed IGF2BP1 lactylation, we incubated purified wild-type IGF2BP1 with purified KAT2A in reaction buffer (25 mM Tris-HCl (pH 7.5), 5 mM beta-glycerophosphate, 2 mM dithiothreitol (DTT), 0.1 mM Na_3_VO_4_, 10 mM MgCl_2_) and 100 nM lactyl-CoA at 30 °C for 30 min. IGF2BP1 lactylation was assessed via immunoblotting.

### Statistical analysis

All data were presented as mean ± standard error of mean (SEM). The majority of experiments were repeated two or three times across independent experiments. Other in vitro assays, including ELISA tests, qRT-PCR, IP assays, and vivo experiments, were performed with three biological replicates. For comparisons between two groups, a Student’s *t* test was employed for data with normal distributions and the rank-sum test was used for data with non-normal distributions. For three or more groups, results were analyzed with one-way analysis of variance (ANOVA). A *p* value below 0.05 was considered significant, and significance was indicated as *P < 0.05, **P < 0.01, ***P < 0.001 and ****P < 0.0001.

## Supplementary information

Unprocessed western blots

Supplementary Materials

## Data Availability

All data relevant to the study are included in the article or supplemental materials. Data are available upon reasonable request.

## References

[1] Bray F, Laversanne M, Sung H, Ferlay J, Siegel RL, Soerjomataram I, et al. Global cancer statistics 2022: GLOBOCAN estimates of incidence and mortality worldwide for 36 cancers in 185 countries. CA Cancer J Clin. 2024;74:229–63. 38572751 10.3322/caac.21834

[2] Peters S, Reck M, Smit EF, Mok T, Hellmann MD. How to make the best use of immunotherapy as first-line treatment of advanced/metastatic non-small-cell lung cancer. Ann Oncol : Off J Eur Soc Med Oncol. 2019;30:884–96. 10.1093/annonc/mdz109 30912805

[3] Xia H, Zhang H, Ruan Z, Zhang H, Sun L, Chen H, et al. Neoadjuvant camrelizumab (an anti-PD-1 antibody) plus chemotherapy or apatinib (a VEGFR-2 inhibitor) for initially unresectable stage II-III non-small-cell lung cancer: a multicentre, two-arm, phase 2 exploratory study. Signal Transduct Target Ther. 2024;9:145. 38871690 10.1038/s41392-024-01861-w PMC11176298

[4] Zhang H, Li S, Liu S, Liao Y, Liu H, Yang M, et al. Therapeutic targeting of cell death-immune crosstalk in cancer to rewire the tumor immune microenvironment. Mol cancer. 2025;24:277. 41174733 10.1186/s12943-025-02491-8 PMC12577006

[5] von Locquenghien M, Zwicky P, Xie K, Jaitin DA, Sheban F, Yalin A, et al. Macrophage-targeted immunocytokine leverages myeloid, T, and NK cell synergy for cancer immunotherapy. Cell;2025:7099–117. 10.1016/j.cell.2025.10.030 41265436

[6] Hou Y, Hu S, Liu C, Chen X, Wang Y, Li Y, et al. Beyond CAR-T cells: exploring CAR-NK, CAR-M, and CAR-γδ T strategies in solid tumor immunotherapy. Front Immunol. 2025;16:1675807. 41181137 10.3389/fimmu.2025.1675807 PMC12571916

[7] Li W, Wu F, Zhao S, Shi P, Wang S, Cui D. Correlation between PD-1/PD-L1 expression and polarization in tumor-associated macrophages: a key player in tumor immunotherapy. Cytokine growth factor Rev. 2022;67:49–57. 35871139 10.1016/j.cytogfr.2022.07.004

[8] Hu J, Zhang L, Xia H, Yan Y, Zhu X, Sun F, et al. Tumor microenvironment remodeling after neoadjuvant immunotherapy in non-small cell lung cancer revealed by single-cell RNA sequencing. Genome Med. 2023;15:14. 36869384 10.1186/s13073-023-01164-9 PMC9985263

[9] Yang B, Li L, Shi D, Zhong T, Xiong H. Lactylation and antitumor immunity. Front Immunol. 2025;16:1690068. 41181157 10.3389/fimmu.2025.1690068 PMC12571743

[10] Liu W, Wang Z, Li Z, Li S, Shi X, Xu Y, et al. Lipid metabolic reprogramming in the tumor microenvironment and its mechanistic role in immunosuppressive cells. Front Immunol. 2025;16:1728354. 41312365 10.3389/fimmu.2025.1728354 PMC12647040

[11] Zhang D, Tang Z, Huang H, Zhou G, Cui C, Weng Y, et al. Metabolic regulation of gene expression by histone lactylation. Nature. 2019;574:575–80. 31645732 10.1038/s41586-019-1678-1 PMC6818755

[12] Watson MJ, Vignali PDA, Mullett SJ, Overacre-Delgoffe AE, Peralta RM, Grebinoski S, et al. Metabolic support of tumour-infiltrating regulatory T cells by lactic acid. Nature. 2021;591:645–51. 33589820 10.1038/s41586-020-03045-2 PMC7990682

[13] Apostolova P, Pearce EL. Lactic acid and lactate: revisiting the physiological roles in the tumor microenvironment. Trends Immunol. 2022;43:969–77. 36319537 10.1016/j.it.2022.10.005 PMC10905416

[14] Zhang Y, Song H, Li M, Lu P. Histone lactylation bridges metabolic reprogramming and epigenetic rewiring in driving carcinogenesis: Oncometabolite fuels oncogenic transcription. Clin Transl Med. 2024;14:e1614. 38456209 10.1002/ctm2.1614 PMC10921234

[15] Zaccara S, Ries RJ, Jaffrey SR. Reading, writing and erasing mRNA methylation. Nat Rev Mol cell Biol. 2019;20:608–24. 31520073 10.1038/s41580-019-0168-5

[16] Zhu TY, Hong LL, Ling ZQ. Oncofetal protein IGF2BPs in human cancer: functions, mechanisms and therapeutic potential. Biomark Res. 2023;11:62. 37280679 10.1186/s40364-023-00499-0 PMC10245617

[17] Zhu ZM, Huo FC, Zhang J, Shan HJ, Pei DS. Crosstalk between m6A modification and alternative splicing during cancer progression. Clin Transl Med. 2023;13:e1460. 37850412 10.1002/ctm2.1460 PMC10583157

[18] Zhou H, Sun Q, Feng M, Gao Z, Jia S, Cao L, et al. Regulatory mechanisms and therapeutic implications of insulin-like growth factor 2 mRNA-binding proteins, the emerging crucial m(6)A regulators of tumors. Theranostics. 2023;13:4247–65. 37554271 10.7150/thno.86528 PMC10405845

[19] Yang Y, Li S, To KKW, Zhu S, Wang F, Fu L. Tumor-associated macrophages remodel the suppressive tumor immune microenvironment and targeted therapy for immunotherapy. J Exp Clin cancer Res : CR. 2025;44:145. 40380196 10.1186/s13046-025-03377-9 PMC12083052

[20] Jiang L, Wang YJ, Zhao J, Uehara M, Hou Q, Kasinath V, et al. Direct tumor killing and immunotherapy through anti-SerpinB9 therapy. Cell. 2020;183:1219–1233.e1218. 33242418 10.1016/j.cell.2020.10.045 PMC7927154

[21] Liu B, Li W, Zhang W, Feng C, Wan L, He S, et al. PKMYT1 kinase ameliorates cisplatin sensitivity in osteosarcoma. Signal Transduct Target Ther. 2025;10:165. 40393983 10.1038/s41392-025-02250-7 PMC12092789

[22] Wang Q, Liu T, Fang Y, Xie S, Huang X, Mahmood R, et al. BUBR1 deficiency results in abnormal megakaryopoiesis. Blood 2004;103:1278-85. 10.1182/blood-2003-06-2158 14576056

[23] Xu C, Fillmore CM, Koyama S, Wu H, Zhao Y, Chen Z, et al. Loss of Lkb1 and Pten leads to lung squamous cell carcinoma with elevated PD-L1 expression. Cancer cell. 2014;25:590–604. 24794706 10.1016/j.ccr.2014.03.033 PMC4112370

[24] Zhang Y, Ma J, Li P, Lu K, Han Y, Hu X, et al. Fatty acid metabolism shapes immune responses in chronic lymphocytic leukemia. Biomark Res. 2025;13:42. 40075418 10.1186/s40364-025-00753-7 PMC11905569

[25] Shu J, Jiang J, Zhao G. Identification of novel gene signature for lung adenocarcinoma by machine learning to predict immunotherapy and prognosis. Front Immunol. 2023;14:1177847. 37583701 10.3389/fimmu.2023.1177847 PMC10424935

[26] Korbecki J, Kojder K, Kapczuk P, Kupnicka P, Gawrońska-Szklarz B, Gutowska I, et al. The effect of hypoxia on the expression of CXC chemokines and CXC chemokine receptors-a review of literature. Int J Mol Sci. 2021;22:843. 10.3390/ijms22020843 PMC7830156 33467722

[27] Zhao Z, Qin J, Qian Y, Huang C, Liu X, Wang N, et al. FFAR2 expressing myeloid-derived suppressor cells drive cancer immunoevasion. J Hematol Oncol. 2024;17:9. 38402237 10.1186/s13045-024-01529-6 PMC10894476

[28] Schiwon M, Weisheit C, Franken L, Gutweiler S, Dixit A, Meyer-Schwesinger C, et al. Crosstalk between sentinel and helper macrophages permits neutrophil migration into infected uroepithelium. Cell. 2014;156:456–68. 24485454 10.1016/j.cell.2014.01.006 PMC4258064

[29] Tian F, Huang J, Fan W, Li X, Zhan Y, Zhu K, et al. m(6)A-modified EHD1 controls PD-L1 endosomal trafficking to modulate immune evasion and immunotherapy responses in lung adenocarcinoma. Cancer Commun (Lond, Engl). 2025;45:1285–308. 10.1002/cac2.70052 PMC12531423 40703029

[30] Wang S, Zeng Y, Zhu L, Zhang M, Zhou L, Yang W, et al. The N6-methyladenosine Epitranscriptomic Landscape of Lung Adenocarcinoma. Cancer Discov. 2024;14:2279–99. 38922581 10.1158/2159-8290.CD-23-1212 PMC11528209

[31] Bell HN, Zou W. Beyond the barrier: unraveling the mechanisms of immunotherapy resistance. Annu Rev Immunol. 2024;42:521–50. 38382538 10.1146/annurev-immunol-101819-024752 PMC11213679

[32] Zhang CX, Huang RY, Sheng G, Thiery JP. Epithelial-mesenchymal transition. Cell. 2025;188:5436–86. 41043405 10.1016/j.cell.2025.08.033

[33] Chen AN, Luo Y, Yang YH, Fu JT, Geng XM, Shi JP, et al. Lactylation, a novel metabolic reprogramming code: current status and prospects. Front Immunol. 2021;12:688910. 34177945 10.3389/fimmu.2021.688910 PMC8222712

[34] Li C, Liu Z, Kong D, Li Z, Li L. Lactylation: a novel driver of drug resistance in the tumor microenvironment. Cancer Drug Resist. 2025;8:39. 40843350 10.20517/cdr.2025.90 PMC12366433

[35] Zhu W, Fan C, Hou Y, Zhang Y. Lactylation in tumor microenvironment and immunotherapy resistance: new mechanisms and challenges. Cancer Lett. 2025;627:217835. 40447130 10.1016/j.canlet.2025.217835

[36] Yin C, Zhang C, Wang Y, Liu G, Wang N, Liang N, et al. ALDOB/KAT2A interactions epigenetically modulate TGF-β expression and T cell functions in hepatocellular carcinogenesis. Hepatol (Baltim, Md). 2025;81:77–93. 10.1097/HEP.0000000000000704 38051951

[37] Zhu R, Ye X, Lu X, Xiao L, Yuan M, Zhao H, et al. ACSS2 acts as a lactyl-CoA synthetase and couples KAT2A to function as a lactyltransferase for histone lactylation and tumor immune evasion. Cell Metab. 2025;37:361–376.e367. 39561764 10.1016/j.cmet.2024.10.015

[38] Zheng B, Pan Y, Qian F, Liu D, Ye D, Yu B, et al. High sugar induced RCC2 lactylation drives breast cancer tumorigenicity through upregulating MAD2L1. Adv Sci. 2025;12:e2415530. 10.1002/advs.202415530 PMC12140329 40145796

[39] Zhang Y, Gao Y, Ding Y, Jiang Y, Chen H, Zhan Z, et al. Targeting KAT2A inhibits inflammatory macrophage activation and rheumatoid arthritis through epigenetic and metabolic reprogramming. MedComm. 2023;4:e306. 37313329 10.1002/mco2.306 PMC10258526

[40] Kou R, Li T, Fu C, Jiang D, Wang Y, Meng J, et al. Exosome-shuttled FTO from BM-MSCs contributes to cancer malignancy and chemoresistance in acute myeloid leukemia by inducing m6A-demethylation: a nano-based investigation. Environ Res. 2024;244:117783. 38048862 10.1016/j.envres.2023.117783

[41] Shi J, Zhang Q, Yin X, Ye J, Gao S, Chen C, et al. Stabilization of IGF2BP1 by USP10 promotes breast cancer metastasis via CPT1A in an m6A-dependent manner. Int J Biol Sci. 2023;19:449–64. 36632454 10.7150/ijbs.76798 PMC9830507

[42] Wallis N, Gershon T, Shaaby S, Oberman F, Grunewald M, Duran D, et al. AVJ16 inhibits lung carcinoma by targeting IGF2BP1. Oncogene. 2025;44:3239–54. 40629079 10.1038/s41388-025-03449-2 PMC12375500

[43] Yin N, Liu Y, Weems C, Shreeder B, Lou Y, Knutson KL, et al. Protein kinase Cι mediates immunosuppression in lung adenocarcinoma. Sci Transl Med. 2022;14:eabq5931. 36383684 10.1126/scitranslmed.abq5931 PMC11457891

[44] Wang X, He J, Ding G, Tang Y, Wang Q. Overcoming resistance to PD-1 and CTLA-4 blockade mechanisms and therapeutic strategies. Front Immunol. 2025;16:1688699. 41112253 10.3389/fimmu.2025.1688699 PMC12531160

[45] Liu NN, Yi CX, Wei LQ, Zhou JA, Jiang T, Hu CC, et al. The intratumor mycobiome promotes lung cancer progression via myeloid-derived suppressor cells. Cancer cell. 2023;41:1927–1944.e1929. 37738973 10.1016/j.ccell.2023.08.012

[46] Wu X, Li B, Liao H, Chen X, Yu X, Hu J, et al. Nanodrug modulates premetastatic niche and suppresses metastatic lung adenocarcinoma via programmed cell death ligand 1 blockade and STING pathway activation. ACS Nano. 2025;19:23893–907. 40554773 10.1021/acsnano.5c05274

[47] Yan H, Huang J, Wang Y, Zhang Y, Ren W, Zhai Z, et al. Berbamine as potential STING inhibitor For KRAS-mutant non-small cell lung cancer. Pharmacol Res. 2025;216:107777. 40383171 10.1016/j.phrs.2025.107777

[48] Li G, Li Q, Yang S, Guo D, Tao Y, Jia Y. FGA modulates immune infiltration and tumor progression via SLC7A11/xCT-mediated disulfidptosis in the tumor microenvironment of lung adenocarcinoma. Front Immunol. 2025;16:1595900. 40861466 10.3389/fimmu.2025.1595900 PMC12375581

[49] Wang H, Peng H, Zhang Z, Abulimiti Y, Hu J, Zhou Y, et al. FTO-mediated m(6)A demethylation regulates IGFBP3 expression and AKT activation through IMP3-dependent P-body re-localisation in lung cancer. Clin Transl Med. 2025;15:e70392. 40621649 10.1002/ctm2.70392 PMC12230637

