# Supplementary material for: KAT2A-IGF2BP1-CXCL2 axis in the high lactate tumor microenvironment facilitates resistance to anti-PD-1 therapy in lung adenocarcinoma by recruiting myeloid-derived suppressor cells
Source: Cell Death Dis. 2026 Jun 25;17(1):790. doi: 10.1038/s41419-026-09018-z (PMC13558772; doi:10.1038/s41419-026-09018-z)
Supplement: Supplementary file 1 [file 41419_2026_9018_MOESM2_ESM.docx]

**Supplementary Materials**

**Supplementary Figure 1**

(A) Prognostic analysis of KAT2A by ROC curve (AUC = 0.612) in TCGA-LUAD.

(B) Assessment of lactylation levels in pre-treatment biopsy specimens reveals no significant difference between tumors that subsequently responded to therapy and those that developed resistance.

(C) Comparative analysis of immune cell infiltration between KAT2A high or low expression clusters in LUAD Patients.

(D) IGF2BP1 prognostic performance in TCGA-LUAD evaluated by ROC curve analysis across 1-, 3-, and 5-year timepoints.

(E) Kaplan–Meier survival analysis for LUAD patients grouped into IGF2BP1 high or low expression determined by the comparison with the median. p = 0.012 by log-rank test.

(F) Immunoprecipitation (IP) of PC-9 cell lysates was performed using anti-Pan-Kla antibody-conjugated beads to enrich for lactylated proteins, and subsequently with anti-IGF2BP1 antibody-conjugated beads, followed by Western blot analysis to detect Pan-Kla or IGF2BP1 signals.

(G, H) Molecular docking analysis of the interaction between KAT2A and IGF2BP1 proteins using HDOCK.

(I) In vitro measurement of CXCL2 protein levels by ELISA in A549 cells.

**Supplementary Table 1**

Prime sequence

| Gene | Forward (5’-3’) | Reverse (5’-3’) |
| --- | --- | --- |
| CD8A | TGAACTCTACTACTACCAA | CTGAGCCACGGGGCCGACAA |
| CD69 | TGGGCAAGTACAATTGCCCAG | TGTGGTGGAGAAGAAGTAAC |
| CD25 | CACTGCAGGGAGCCACCTTCC | AGGACCTCTCTGTAGAGCCT |
| CXCL2 | GAGCGCCCCTGGCCACTGAA | ATGACTTCGGTTTGGGCGCA |
| CXCL2 site1 | CGACTGGAGCTGTTGGTCAG | TAACCTTAATGGCAACTTTGTG |
| CXCL2 site2 | TATTAATCTGTTCTGGTGCCAG | AGCACAACTGGCAAACTCCAAT |
| CXCL2 site3 | AGAGACAATATTATTTAGTTTA | AGAGCCTATTGCTCTTTGTCA |
| CXCL2 site4 | TTACAGTGGCAAATCCAACTG | AGGTGGCCTCTGCAGCTGTG |
| CXCL2 site5 | ATGACATATCACATGTCAGC | CATCTTTACAGTTACAAAAT |
